# Novel Vaccine Technologies in Veterinary Medicine: A Herald to Human Medicine Vaccines

**DOI:** 10.3389/fvets.2021.654289

**Published:** 2021-04-15

**Authors:** Virginia Aida, Vasilis C. Pliasas, Peter J. Neasham, J. Fletcher North, Kirklin L. McWhorter, Sheniqua R. Glover, Constantinos S. Kyriakis

**Affiliations:** ^1^Department of Pathobiology, College of Veterinary Medicine, Auburn University, Auburn, AL, United States; ^2^Emory-University of Georgia (UGA) Center of Excellence for Influenza Research and Surveillance (CEIRS), Auburn, AL, United States; ^3^Department of Chemistry, Emory University, Atlanta, GA, United States; ^4^Center for Vaccines and Immunology, University of Georgia, Athens, GA, United States

**Keywords:** veterinary vaccines, new technology vaccines, food animals, companion animals, infectious diseases, disease control and prevention

## Abstract

The success of inactivated and live-attenuated vaccines has enhanced livestock productivity, promoted food security, and attenuated the morbidity and mortality of several human, animal, and zoonotic diseases. However, these traditional vaccine technologies are not without fault. The efficacy of inactivated vaccines can be suboptimal with particular pathogens and safety concerns arise with live-attenuated vaccines. Additionally, the rate of emerging infectious diseases continues to increase and with that the need to quickly deploy new vaccines. Unfortunately, first generation vaccines are not conducive to such urgencies. Within the last three decades, veterinary medicine has spearheaded the advancement in novel vaccine development to circumvent several of the flaws associated with classical vaccines. These third generation vaccines, including DNA, RNA and recombinant viral-vector vaccines, induce both humoral and cellular immune response, are economically manufactured, safe to use, and can be utilized to differentiate infected from vaccinated animals. The present article offers a review of commercially available novel vaccine technologies currently utilized in companion animal, food animal, and wildlife disease control.

## Introduction

From Edward Jenner and Louis Pasteur in the eighteenth and nineteenth centuries, to the eradication of rinderpest in bovine and smallpox in the human populations by the twentieth century, vaccines have played a pivotal role in the survival, health, and general well-being of humans and animals (1–3).

The ultimate goal of vaccination is to generate humoral and/or cell-mediated immunity thereby inducing the production of immunological memory that confers protection against subsequent natural infection(s). The elicitation of neutralizing antibodies has long been the major goal of vaccines, however in addition to neutralizing antibodies, T-cell mediated immune responses have been shown to be crucial for effective protection against pathogens such as varicella virus, HIV, tuberculosis, and malaria (4–9).

The adaptive immune response is activated primarily through the presentation of antigens bound to a Major Histocompatibility Complex (MHC) I or II on the surface of antigen presenting cells (APCs) to T-cells and B-cells within secondary lymphoid organs. However, B-cells can take up particulate and antigen without the help of APCs provided the antigen is small enough (10). MHC-I is found in all nucleated cells while MHC-II is exclusively expressed by dendritic cells, macrophages, monocytes, B-cells, and mucosal epithelial cells (11). Nonetheless, because T cells are unable to directly interact with antigen, the mechanism of MHC presentation in conjunction with appropriate signaling plays a pivotal role in the effector cells activated and is particularly important in vaccine development in which a T-cell mediated response is desired (12). The MHC presentation is dependent on the intracellular location of the antigen processing. Cytosol derived-antigens, such as in the case of virally infected somatic cells, are processed onto MHC-I complexes and interact with CD8+ T cells, also known as cytotoxic T cells (CTLs) which directly kill infected cells (13). APCs can also present exogenously acquired antigens on MHC-I complexes, a process termed cross-presentation (14) and upon migration to lymph nodes, will activate CTLs which will migrate out of the lymph node to eliminate infected cells.

Exogenous antigens acquired by endocytosis are presented on MHC-II molecules and interact with CD4+T helper (T_H_) cells. T-helper cells have various fates and effector functions which are influenced by the type of signal elicited during priming and activation. Pertinent to vaccine production, T-helper 1 (T_H_1) cells produce interferon-γ and tumor necrosis factor alpha which potentiate the effector function of phagocytes and increase inflammation (15). Thus, vaccine-induced memory T_H_1 cells are particularly sought for intracellular pathogens. T-helper 2 (T_H_2) cells facilitate B-cell proliferation whilst antagonizing T_H_1 differentiation and are therefore associated with increased humoral responses and of particular interest for vaccines targeting parasites or allergic responses (16, 17). T-follicular helper cells (T_FH_) interact with B-cells that present antigen on MHC-II molecules (12, 18, 19). Only B cells that receive co-stimulatory signals from T_FH_ cells are able to generate high-affinity IgG antibodies or mature into memory B-cells (20). As such, vaccines aimed to generate robust B-cell memory need to also stimulate T-cell responses.

The classical inactivated and modified-live vaccines (IV and MLV, respectively), also known as first generation vaccines, have given humans and animals alike advantages over the pathogenic world that surrounds them. These vaccines have also had an economic impact due to the success that has been seen in livestock industries (21). IVs are safe and relatively inexpensive to produce, predominantly present antigens *via* the MHC-II pathway and mainly induce humoral immune responses. Due to this disadvantage, pathogens requiring a strong cell-mediated response can escape the pressure elicited by the vaccine (22). MLVs circumvent this issue, due to their ability to successfully replicate within the host and elicit protective immunity against their respective pathogens. These attenuated pathogens mimic natural infection thereby eliciting both MHC-I and MHC-II pathways. Some MLVs have been shown to elicit mucosal IgA antibodies, a unique feature to only a handful of vaccines administered *via* the oral or nasal route (23). However, MLVs pose a slight risk to animals as there were rare cases where attenuated strains regained pathogenicity, causing the spread of disease (21, 24–27). Additionally, MLVs are contraindicated in severely immunocompromised individuals due to the risk of disease (28). These classical vaccines have predominated commercial human and animal immunizations for the past 100 years. However, the aforementioned disadvantages have directed second and third-generation vaccines into the limelight of exploration.

**Figure 1:**
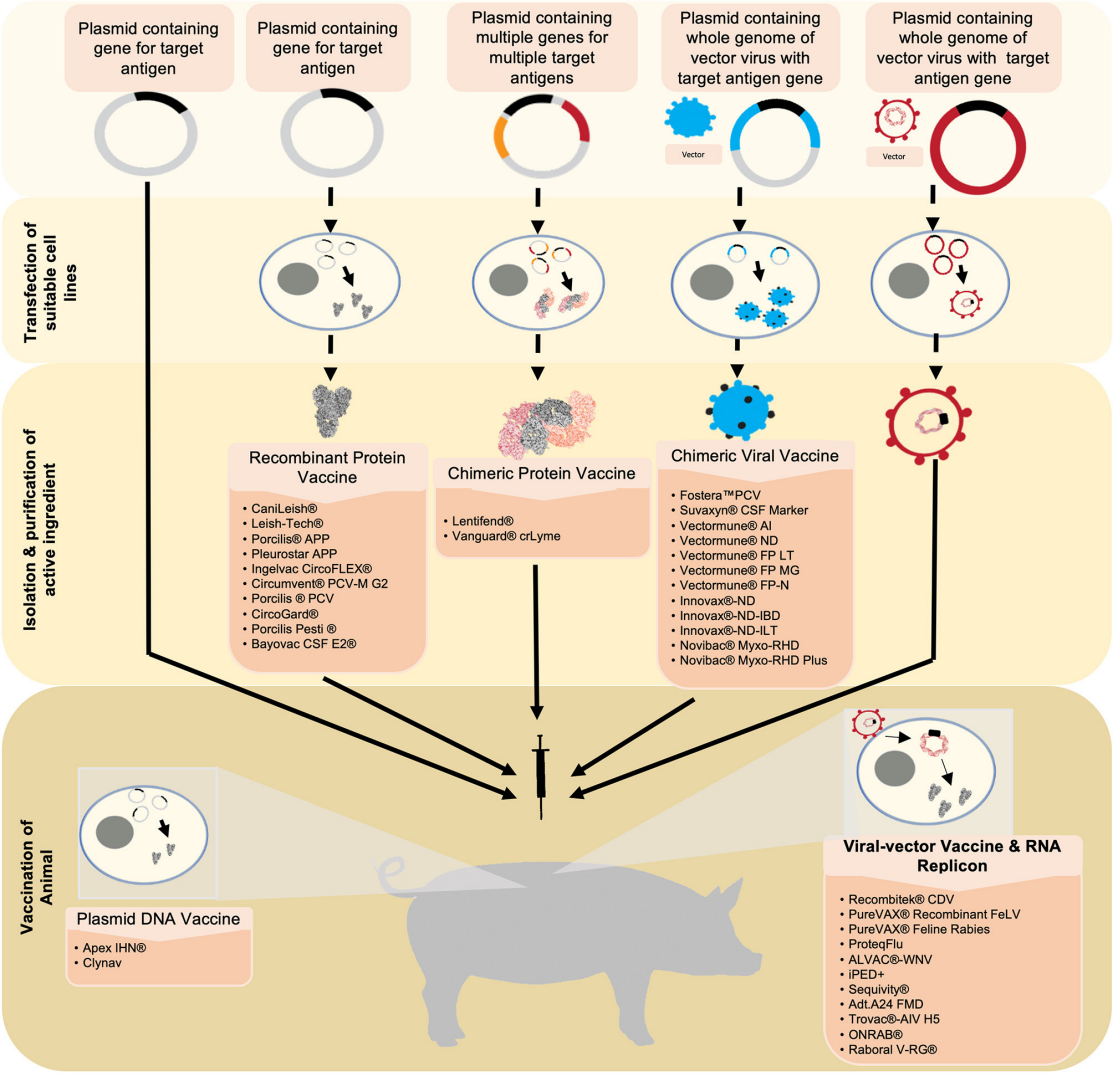
Six novel vaccine technologies discussed in this review are simplified and summarized starting from the generation and production of antigens to the vaccination. Beginning with plasmid-DNA vaccines, the target antigen is inserted into a plasmid. This serves as the active ingredient that will be used to vaccinate the animal. Upon vaccination, the plasmid-DNA vaccine carrying the DNA encoding for the target antigen is translated into the desired protein in the vaccine recipient's cells. The antigen is then expressed from the cell, consequently eliciting an immune response. Recombinant protein vaccines and chimeric protein vaccines utilize a similar technology. However, suitable cell-lines are transfected with the plasmid in which the antigen(s) is/are expressed. The antigen(s) is/are then harvested, purified, and formulated into the vaccine. Chimeric viral vaccines utilize a plasmid containing the whole genome of a virus that will be used as a vector in addition to the target gene for the desired antigen. This plasmid is then used to transfect a suitable cell-line in which a whole virus expressing the integrated antigen is produced. This virus is harvested and purified, and formulated into a vaccine. Viral vectors utilize a virus that has been engineered to express the gene of interest. The virus is formulated into a vaccine and will release the recombinant genes into the host cells. Similar to a plasmid-DNA vaccine, the genes will be transcribed into the target antigen which will then be expressed and elicit an immune response. RNA replicon vaccines utilize a RNA segment that encodes the desired antigens encapsulated in a vesicle carrier. Once in the host's cell, the RNA is directly translated, resulting in the expression of the target antigen.

These second and third generation vaccines have shown success in veterinary medicine thereby paving the way for advancement in human medicine (Figure 1). Second generation vaccines include subunit elements, conjugated/recombinant antigens, or synthetic proteins (Table 1). Recombinant subunit vaccines do not use virus (inactivated or live), but rather utilize antigen production through overexpression and purification of the antigen. This can be achieved through multiple routes, including the baculovirus expression vector system (BEVS). Subunit vaccines oftentimes lack the pathogen associated molecular patterns that the immune system utilizes to recognize pathogens *via* pattern recognition receptors. Because of this, subunit vaccines necessitate adjuvants with co-stimulatory activity that enhance the magnitude and quality of the immune response. Furthermore, these types of vaccines are generally recognized by antigen presenting cells *via* the intravesicular route and are consequently presented on MHC-II complexes.

**Table 1:** Subunit and recombinant protein vaccines.

**Species**	**Vaccine**	**Manufacturer**	**Pathogen**	**Technology**
Canine	CaniLeish® (LiESP/QA-21)	Virbac	Leishmania	Subunit (Cell-free, serum-free culture system)
	Leish-Tech®	CEVA Animal Health	Leishmania	Recombinant Protein
	Lentifend®	Laboratorios Leti	Leishmania	Chimeric protein
	Vanguard® crLyme	Zoetis	Borrelia burgdorferi	Chimeric Protein
Swine	Porcilis®APP	Merck Animal Health	A.pleuropneumoniae	Subunit
	Pleurostar APP	Novartis	A.pleuropneumoniae	Subunit
	Ingelvac CircoFLEX®	Boehringer Ingelheim	Porcine Circovirus Type 2	Subunit (BEVS)
	Circumvent® PCV-M G2	Merck Animal Health	Porcine Circovirus Type 2	Subunit (BEVS)
	Procilis® PCV	Merck Animal Health	Porcine Circovirus Type 2	Subunit (BEVS)
	CircoGard	Pharmgate Animal Health	Porcine Circovirus Type 2	Subunit (BEVS)
	Porcilis Pesti®	Merck	Classical Swine Fever	Subunit (BEVS)
	Bayovac CSF E2®	Bayer	Classical Swine Fever	Subunit (BEVS)

Third generation vaccines include gene-based (DNA and RNA) vaccines, viral-vector platforms, and live or inactivated chimeric vaccines. DNA and RNA-based vaccines is a fundamentally new approach to vaccination, involving the use of plasmid DNA delivered through injection (Table 2). Advancements in molecular biology techniques have allowed us to manipulate these polynucleotides to our advantage, providing alternative routes to the classical vaccine technologies (29, 30). DNA vaccines employ the use of a plasmid containing the DNA encoding the antigen(s) of interest. Once inserted into host cells, the cellular machinery will express the antigens encoded by the DNA and an immune response will be elicited. Some advantages of DNA vaccines include the safe administration to immunocompromised individuals compared to MLVs, the potential for combining multiple plasmids for a broad-spectrum combination vaccine, and the ease of engineering compared to classical vaccines (31–33). Along with these advantages DNA vaccines induce both humoral and cell mediated responses, and function as pathogen associated molecular patterns (PAMPS) attenuating the necessity for adjuvant (32, 34, 35). More specifically, plasmid-DNA and RNA vaccines transfect cells and thus mimic intracellular pathogen protein production and typically induce strong MHC-I mediated CD8+T cell responses (36). Transfected somatic cells will present antigen on MHC-I, thereby eliciting CTLs cross-primed by dendritic cells. Additionally, APCs engulf transfected cells and present antigen on MHC-II complexes to elicit a CD4+ T-cell response (37).

**Table 2:** DNA vaccines.

**Species**	**Vaccines**	**Manufacturer**	**Pathogen**	**Plasmid(s)**
Salmonid	Apex IHN ®	Elanco (Aqua Health)	Infectious Hematopoietic Necrosis	pUK21-A2, pUK-ihnG
	Clynav	Elanco (Aqua Health)	Salmonid Alphavirus Subtype 3	PUK-SPDV-poly2#1

Recombinant viral vector vaccines are novel technologies in veterinary medicine that utilize viruses as tools for vaccinology (Table 3). These vaccines are genetically engineered and involve the insertion of DNA encoding key antigens into a viral vector. The safety profile is similar to inactivated (killed) subunit vaccines and stimulate both cell-mediated, specifically CD8+T cell responses, and humoral immune responses (9, 38). Pox viral vectors were the first to be studied and established in the 1980's, with various backbones being utilized to induce responses to various animal pathogens, including canarypox and fowlpox backbones (39–43). Adenovirus vectors have been explored as systems of treatments for numerous infections, and even as vaccines against tumor-associated antigens (44). Positive sense RNA-containing alphaviruses have also been used as vector backbones, and these constructs include two types: full-length infectious clones and replicon vectors. The latter type is advantageous due to their lack of structural protein genes, only containing the non-structural genomic region and the genes encoding the antigen(s) of interest (45). For alphavirus-replicons, foreign genes of interest can be inserted in the place of the structural genes generating self-replicating RNA replicons (RP) (46). Upon inoculation, the RP is engulfed by dendritic cells and consequently directs the translation of large amounts of protein in the cells resulting in the presentation of the antigen. This essentially makes them self-replicating RNA molecules. These concepts can then be extended into chimeric recombinant vector vaccines, where the principles are the same, yet the genes, and by extension antigens, of interest are taken from multiple types of the pathogen and placed within the same vector, aiming to elicit a broader immune response.

**Table 3:** Recombinant viral vector vaccines.

**Species**	**Vaccine**	**Manufacturer**	**Pathogen**	**Technology (viral-vector)**
Canine	Recombitek® CDV	Boehringer Ingelheim	Canine Distemper Virus	Viral-Vector (canarypox)
Feline	PureVAX® Recombinant FeLV	Boehringer Ingelheim	Feline Leukemia Virus	Viral-Vector (canarypox)
	PureVAX® Feline Rabies	Boehringer Ingelheim	Rabies	Viral-Vector (canarypox)
Equine	ProteqFlu	Boehringer Ingelheim	Equine Influenza	Viral-Vector (canarypox)
	ALVAC®-WNV	Pfizer	West Nile Virus	Viral-Vector (canarypox)
Swine	Fostera™PCV	Zoetis	Porcine Circovirus Type 2	Chimeric Viral-vector (PCV-1)
	Suvaxyn® CSF Marker	Zoetis	Classical Swine Fever virus	Chimeric Viral-vector (BVDV)
	iPED+	Merck Animal Health	Porcine Endemic Diarrhea virus	RNA Replicon (VEEV)
	Sequivity®	Merck Animal Health	Swine influenza A virus	RNA Replicon (VEEV)
Bovine	Adt.A24 FMD	GenVec	Foot and Mouth Disease	Viral-vector (adenovirus)
Avian	Trovac®-AIV H5	Boehringer Ingelheim	Avian Influenza	Viral-vector (fowlpox)
	Vectormune® AI	CEVA Biomune	Avian Influenza	Chimeric Viral-vector (HVT/MD)
	Vectormune® ND	CEVA Biomune	Newcastle Disease	Chimeric Viral-vector (HVT/MD)
	Vectormune® FP LT	CEVA Biomune	Infectious Laryngotracheitis virus	Chimeric Viral-vector (fowlpox)
	Vectormune® FP MG	CEVA Biomune	Mycoplasma Gallisepticum	Chimeric Viral-vector (fowlpox)
	Vectormune® FP-N	CEVA Biomune	Newcastle Disease	Chimeric Viral-vector (fowlpox)
	Innovax®-ND	Merck Animal Health	Newcastle Disease	Chimeric Viral-vector (HVT/MD)
	Innovax®-ND-IBD	Merck Animal Health	Newcastle disease and Infectious bursal disease	Chimeric Viral-vector (HVT/MD)
	Innovax®-ND-ILT	Merck Animal Health	Newcastle disease and infectious laryngotracheitis	Chimeric Viral-vector (HVT/MD)
Wildlife	ORNAB®	Artemis Technologies, Inc.,	Rabies	Viral-vector (human adenovirus type 5)
	Raboral V-RG®	Boehringer Ingelheim	Rabies	Viral-vector (vaccinia virus)
Rabbits	Novibac® Myxo-RHD	Merck Animal Health	Rabbit Hemorrhagic Disease	Chimeric Viral-vector (myxoma virus)
	Novibac® Myxo-RHD Plus	Merck Animal Health	Rabbit Hemorrhagic Disease	Chimeric Viral-vector (myxoma virus)

Another component that distinguishes veterinary from human vaccines is the technology that enables the differentiation of infected and vaccinated animals (DIVA), making them a critical tool in disease control and eradication (47). This technology has also made a huge impact on imports and exports as it provides a sensitive, rapid, and inexpensive method for determining pathogen free flocks and herds (48). Most DIVA vaccines, or marker vaccines, are based on recombinant deletion mutants of wild-type pathogens, where gene segments expressing viral proteins, such as the herpesvirus envelope glycoprotein (gE), have been removed. Other DIVA vaccines are based on subunit vaccines and inactivated whole virus vaccines (Table 4). Because DIVA vaccines elicit a different immune response from that elicited by a natural infection companion diagnostic tests, typically an enzyme linked immunosorbent assays (ELISA) can be utilized to discern those infected and those vaccinated. DIVA vaccines have been utilized in the control of Foot-and-mouth disease, Classical swine fever, Bovine rhinotracheitis, and the eradication of Pseudorabies (Aujesky's disease) in pigs (49–51).

**Table 4:** DIVA vaccines.

**Species**	**Vaccine**	**Manufacturer**	**Pathogen**
Canine	Leish-Tech®	CEVA Animal Health	Leishmania
	Lentifend®	Laboratorios Leti	Leishmania
Feline	PureVAX® Recombinant FeLV	Boehringer Ingelheim	Feline Leukemia Virus
Swine	Porcilis® Begonia	Merck Animal Health	Suid Herpesvirus 1
	Auskipra® GN	Hipra	Suid Herpesvirus 1
	Suvaxyn® CSF Marker	Zoetis	Classical Swine Fever Virus
Bovine	Adt.A24 FMD	GenVec	Foot and Mouth Disease
	Bovilis® IBR Marker Live	Intervet	Bovine Herpesvirus-1
	Hiprabovis® IBR Marker Live	Hipra	Bovine Herpesvirus-1
	Bayovac IBR Marker Vivum	Bayer	Bovine Herpesvirus-1
	Bayovac IBR Marker Inactivum	Bayer	Bovine Herpesvirus-1
	Rispoval® IBR-Marker Inactivated	Zoetis	Bovine Herpesvirus-1
	Rispoval® IBR-Marker Live	Zoetis	Bovine Herpesvirus-1

Overall, veterinary medicine has made great strides in vaccine development for a wide array of pathogens, and has spearheaded vaccinology methodologies and designs, being years in advance compared to human vaccine technologies. In this review, current commercially available and licensed technologies being utilized in veterinary vaccinology are presented.

## Companion Animals

### Canine Vaccines

#### Canine Distemper Virus—Recombitek® Combination Vaccines (CDV)

Canine distemper virus (CDV) belongs to the *Paramyxoviridae* family and is closely related to the human measles virus and bovine rinderpest virus. CDV is found worldwide, affects all members of the *canidae* family, and is responsible for significant disease, often resulting in high morbidity and mortality in unprotected animals. Recombitek® vaccines, produced by Merial Animal Health (now Boehringer Ingelheim Animal Health), utilize a recombinant canarypox-vector expressing both the antigenic hemagglutinin and fusion glycoproteins of CDV and are co-formulated with other MLVs (adenovirus type 2, coronavirus, parainfluenza, or parvovirus) or bacterial antigens. These vaccines are the only virus-vectored CDV vaccines licensed and commercially available for canines to-date. One of the major benefits to this vaccine is the canarypox-vector does not have the complete CDV genome nor infectious components of CDV and therefore the risk of post-vaccinal CDV encephalitis is eliminated (52). Studies have shown the Recombitek® CDV has comparable time-to-immunity to MLV-CDV vaccines, can confer moderate protection against virus challenge within hours of being vaccinated, and fully protects animals within 1 week of vaccination (53). Furthermore, unlike MLV-CDV vaccines, Recombitek® CDV can be utilized in pre-weaning disease and immunosuppressed individuals as it was shown to protect puppies in the presence of maternal antibodies whilst not suppressing lymphocyte responsiveness (54, 55). Recombitek® CDV has a significant anamnestic response and confers a 4-fold greater increase in titer upon booster vaccination (particularly when the dogs received a MLV-CDV vaccine initially) and a 36 months serologic duration of immunity (56–58). In comparison to MLV-CDV vaccines, Recombitek® CDV induces a lower serum-neutralizing titer compared to MLV-CDV vaccines in non-domestic carnivores (59).

#### Canine Lyme disease—VANGUARD® crLyme

Lyme disease, caused by the spirochete *Borrelia burgdorferi*, is the most common vector-borne illness in North America and Europe and infects a range of vertebrate animals including small mammals, lizards, and birds (3, 60). Previous studies have shown that 63% of dogs exposed to infected ticks, the vector transmitting *B. burgdorferi*, develop clinical signs of Lyme disease which consist of severe elbow or shoulder joint lameness of sudden onset, joint swelling of the shoulder, elbow and carpus, and acute arthritis (61). There are several commercially available canine vaccines against *B. burgdorferi* by inducing the production of outer surface protein A (OspA) borreliacidal antibodies. These antibodies form a membrane attack complex within the tick transmitting *B. burgdorferi* during the blood meal on the host (62). Because OspA is genospecific, it has been identified that targeting both OspA and outer surface protein C (OspC) is a more advantageous vaccination tactic because OspC is conserved among several of the pathogenic *Borrelia* genospecies. Nonetheless, the combination of both antigens provides complete protection from Lyme disease (63–66). VANGUARD® crLyme, created by Zoetis, is the only commercially available chimeric recombinant Lyme vaccine based on chimeric epitope-based recombinant proteins. It contains both antigens for OspA and 14 different linear epitopes derived from seven types of OspC and thus provides broad-spectrum protection (67). While investigating the efficacy and safety of VANGUARD® crLyme, researchers found the vaccine showed a 93.7% reduced incidence of *B. burgdorferi* infection and demonstrated significant humoral responses to both OspA and OspC after vaccination. Upon challenge with ticks suspected of carrying *B. burgdorferi*, vaccinated animals showed no humoral response to OspC antigen suggesting VANGUARD® crLyme prevented *B. burgdorferi* transmission from infected ticks to vaccinated dogs (68). In contrast to these findings, the comparison of VANGUARD® crLyme to Recombitek® Lyme (the commercially available monovalent recombinant OspA vaccine) revealed VANGUARD® crLyme elicited a slower anti-OspA antibody response, had a lower serum borreliacidal activity at all post-vaccination time points, and had inferior immunogenicity (69). Grosenbaugh et al. (69), note that the variation in efficacy could be contributed to the lipidation differences of the antigens but also a mismatch between the OspC antigens used in the vaccine and the antibody assay used to evaluate the response. In a more recent study, VANGUARD® crLyme was shown to induce broadly cross-reactive antibodies to 25 recombinant OspC variants screened against sera of vaccinated animals, significantly reduce histopathological changes at the tick bite site, and prevent *B. burgdorferi*-induced synovitis and dermatitis (68).

#### Canine Visceral Leishmaniasis—CaniLeish®, Leish-Tec®, Letifend®

Canine leishmaniosis (CanL), caused by the protozoan *Leishmania infantum*, is a severe and chronic disease transmitted by the bite of a sandfly. Currently, leishmaniasis is endemic in the Mediterranean basin, Middle East, Central Asia, and Latin America. Importantly, domestic dogs are reservoirs for human visceral leishmaniasis in many areas (70). It is estimated that 30% of dogs in endemic areas are seropositive and some will eventually become clinically ill. Unfortunately, CanL cannot be easily cured with current therapies. Accordingly, the high prevalence implores the creation of an effective vaccine that elicits a robust and long-lasting Th1-mediated response in order to prevent the development of disease after infection. There are three vaccines available on the market to date: CaniLeish® (Virbac S.A.), Leish-Tec® (CEVA animal health), and Lentifend® (Laboratorios Leti). Leishmune® (Zoetis) was removed from the market in 2018 and will therefore not be discussed in this review.

CaniLeish® (LiESP/QA-21) was the first leishmaniasis vaccine in Europe and is indicated for the active immunization of *Leishmania* by providing a significant reduction in disease progression (71). Overall, CaniLeish® is a well-tolerated vaccine formulated with *L. infantum* Excreted-Secreted Protein (LiESP) antigens and a purified extract of *Quillaja saponaria* (QA-21) adjuvant (72). CaniLeish® has a 4 week onset of immunity characterized predominately by an IgG-2 response to ESP and a significantly strong cell-mediated Th1-dominated immune profile that remains persistent for a full year after the primary vaccination course (71, 73, 74). In a major clinical trial, CaniLeish® provided a protection of 68.4% in vaccinated animals compared to unvaccinated controls (71). Additionally, vaccinated dogs had lower mean parasite burdens due to the facilitation of a stronger macrophage-induced intracellular parasitic reduction in conjunction with autologous lymphocytes (73, 75). Unfortunately, CaniLeish® does not prevent initial entry and migration of the parasites and does not produce antibodies that can be distinguished from conventional immunofluorescence antibody tests (IFAT) diagnostic testing (71).

Leish-Tec® is licensed as another second generation vaccine in Brazil. This vaccine contains recombinant protein A2 antigens of various Leishmania species and a saponin adjuvant (76, 77). The vaccine is tolerated similarly to CaniLeish®, elicits an anti-A2 IgG1 antibody, IgG2 antibody, and Th1 immune response 1 month after vaccination (78, 79). This vaccination induces a significant reduction in the transmission of *Leishmania* spp. by sandflies that feed on anti-A2 seropositive vaccinated dogs and reduces the risk of disease progression and all-cause mortality in asymptomatic infected dogs (80, 81). In a field trial study, mean seroconversion time and cumulative incidence of infection among immunized dogs was ~18 months and 27%, respectively while unvaccinated mean seroconversion time was ~9 months and 42%, respectively (79). In that same study 43% of the vaccine recipients eventually developed clinical signs rending the efficacy of Leish-Tec® questionable (79). Currently, the Brazilian government advices the culling of all seropositive dogs. Fortunately, Leish-Tec® is considered a DIVA vaccine since the humoral response induced by Leish-Tec® can be detected by A2-ELISA and does not create cross-reacting interference with conventional leishmaniosis serological diagnostic tests (79, 82).

Lentifend® contains the recombinant antigen Protein Q, a chimeric protein formed by the fusion of five antigenic determinants from four Leishmania proteins and is without adjuvant (76). Lentifend® consistently elicits a cellular and humoral immune response characterized by a significant increase in complement system proteins and an early and statistically significant increase of IgG2 antibodies against Protein Q 2 weeks after vaccination (76, 83, 84). Lentifend® has shown to be very well-tolerated, reduce circulating immune complexes, parasite burden, the incidence of clinical signs, and the number of confirmed cases, and have an overall efficacy of 72% (76, 83). Much like Leish-Tec®, Lentifend® falls into the DIVA category (85, 86).

### Feline Vaccines

#### Feline Leukemia Virus- PureVAX® Recombinant FeLV

Feline leukemia virus is an immunosuppressive retrovirus infecting domestic and wild felids. It can be transmitted *via* direct contact or through virus shed in saliva or nasal secretions and affects multiple organ systems. It is estimated that 2.3–3.4% of all cats in North America are affected (87). PureVAX® Recombinant FeLV, produced by Boehringer Ingelheim Animal Health, is a non-adjuvanted canarypox virus-vectored vaccine that contains the mutated envelope, gag, and truncated polymerase protein of the FeLV subtype A/Glasgow-1 strain (88, 89). The immune response elicited by PureVAX® Recombinant FeLV is characterized by the activation of cell-mediated immunity by inducing FeLV-specific T cell response (89–91). Compared to other commercially available vaccines, Recombinant FeLV has similar degrees of protection from persistent viremia and integration of proviral DNA upon virus challenge and a 93% preventive fraction (92). Nonetheless, a 3-year duration of immunity after a prime and boost vaccination protocol has been shown to confer full protection against persistent viremia (93).

#### Feline Rabies—PureVAX® Feline Rabies

Rabies is a zoonotic, progressive neurological, and fatal infection caused by rabies virus. Rabies infection is present throughout the world, responsible for over 60,000 human deaths per year, and affects all warm-blooded animals (94). PUREVAX® Feline Rabies contains the recombinant canarypox virus (vCP65) that expresses the rabies glycoprotein gene. Inoculation of animals with vCP65 demonstrated an appropriate level of foreign gene product expression sufficient enough to induce rabies-specific serum neutralizing antibodies and T-cell responses to protect against lethal rabies virus challenge for up to 3 years (95). PUREVAX® Feline Rabies provides full protection even when co-administered with other feline vaccines illustrating the usefulness in yearly core vaccinations (96). Additionally, because this vaccine lacks an adjuvant, there is excellent local safety and minimal inflammatory reactions since chronic inflammation at the injection site is a risk factor for vaccine-induced fibrosarcomas in felines (97).

### Equine Vaccines

#### Equine Influenza—ProteqFlu

Equine Influenza virus (EIV) is an *Orthomyxovirus* considered to be an important respiratory disease in horses. Equine Influenza has had major economic and welfare implications within the last decade and is particularly difficult to control due to the virus' inclination to readily undergo antigenic drift and shift. Unfortunately, Vaccine mismatch to the circulating strain can contribute to a significantly decreased efficacy in eliciting appropriate host immune response.

ProtequFlu (marketed by Boehringer Ingelheim, formerly Merial Animal Health) contains two modified live canarypox virus recombinants expressing the EIV hemagglutinin (HA) gene of two significantly important strains of circulating EIVs.ProtequFlu has been shown to generate significantly high IgGa and IgGb anti-influenza antibody titers pre-challenge, a long-term 6-month anamnestic IgGa and IgGb protective responses post challenge with several American lineages and induces a specific IFN-y and IL-2 mRNA expression (98, 99). In animals older than 8 months, vaccination has shown to provide protection after a single dose compared to the required two doses of inactivated vaccine and has thus been utilized as a means for emergency response to IEV outbreaks (100, 101). However, some studies found that foals <8 months did not seroconvert until the third immunization suggesting the presence of maternally derived antibodies contributes to this immunization pattern and might influence vaccination protocols (102). Regarding long-term immunity, ProteqFlu-Te® was not as robust as the whole commercial inactivated vaccines, Equilis Prequenza-Te® and Duvaxyn IE-T Plus®, or when ProteqFlu-Te® was combined in a mixed-vaccination protocol which is a common practice in the field (103).

#### West Nile Virus—ALVAC®-WNV & West Nile-Innovator® DNA

West Nile Virus (WNV) is a mosquito-transmitted neurotropic *Flavivirus* causing debilitating and potentially fatal disease found worldwide in birds, humans and horses (the two latter species being the dead-end hosts) (104, 105). Successful vaccination requires both the induction of neutralizing antibodies and cell-mediated immune response including the elicitation of INF-α, INF-β, and significant involvement of the complement system (104, 106, 107). IgM is critically important for the control of acute and early WNV infection followed by the presence of IgG antibodies which confer long-term protection against WNV re-infection (108, 109).

Merial Animal Health (now Boehringer Ingelheim Animal Health) developed ALVAC®-WNV, a canarypox-vectored recombinant chimeric vaccine that expresses the precursor membrane (prM) and envelope (E) genes of WNV derived from the 1999 New York Isolates (110). ALVAC®-WNV induces neutralizing antibodies and prM/E-insert-specific IFN-y+ producing cells against WNV in vaccinated horses and therefore plays a major role in anti-viral clearance (107, 111). ALVAC®-WNV vaccine was shown to be fully protective against virulent WNV challenge *via* mosquito exposure making it exceptionally applicable in the field (112). Additionally, ALVAC®-WNV induces WNV antibodies as early as 7 days, develop protection against viremia as early as 26 days after a single dose, was fully protective against challenge, and elicited an immune response that could be recalled 9 months after appropriate primary vaccination and booster vaccination (107, 112, 113). West Nile-Innovator® DNA, a WNV DNA plasmid-based vaccine, licensed in 2005 by Fort Dodge Animal Health/Pfitzer, contained an unformulated plasmid DNA encoding the prM and E protein of WNV and a MetaStim™ adjuvant (110, 114). This vaccine resulted in a humoral and strong Th1 response however, the vaccine was discontinued by Pfizer (110, 115, 116).

## Food Animals

### Porcine Vaccines

#### Pleuropneumonia—Porcilis® APP and PleuroStar APP

A second generation of subunit vaccines targeting the bacterium *Actinobacillus pleuropneumoniae* (APP) was previously developed by Merck Animal Health and Novartis. APP is the active agent that causes porcine contagious pleuropneumonia disease in swine through the bacterium's ApxI, ApxII, ApxII, and ApxIV toxins (117, 118). Fifteen known serotypes of APP are currently characterized, each that can cause variable pathogenicity (119). The acute form of porcine contagious pleuropneumonia is often fatal by inducing hemolytic and cytotoxic lung damage leading to pleuropneumonia (119). The disease is most severe in piglets 6–22 weeks old, usually before they go to market (119). Consequently, APP is a huge economic burden for the swine industry. Porcilis® APP and PleurostarAPP are commercially available second-generation subunit vaccines that each provides some cross protection against the 15 serotypes of *A. pleuropneumonia* (120–122). The vaccines are based on four or five purified proteins produced by *c* strains. This includes the exotoxins ApxI, ApxII, ApxIII and a 42 kilodalton outer membrane protein for the development of PorcilisAPP, and the ApxII, TbpB, CysL, OmlA, and OmlA proteins for PleurostarAPP (119, 123).

Porcilis® APP has been shown to develop a protective immunity with a peak 2–3 weeks after boost vaccination which can be maintained for up to seven weeks, confer protection in terms of clinical signs, reduced lung lesions, and reduce mortality for serovar 1 (123). In an experiment conducted by Del Pozo Sacristan et al., Porcilis® APP was evaluated in herds chronically affected by pleurisy. Vaccinated animals had significantly lower prevalence and extent of pleurisy 4.1 and 2.5%, respectively vs. the non-vaccinated animals of 18.5 and 8.0%, respectively. Vaccinated animals gained more weight than pigs in the non-vaccinated group. Additionally, antimicrobial use and mortality were reduced in vaccinated animals suggesting that although vaccination may not prevent clinical expression of APP infection, it could be useful in reducing the impact of infection (121, 123).

#### Porcine Circovirus Type 2—Ingelvac CircoFLEX®, Circumvent® PCV-M G2, Porcilis® PCV, CircoGard & Fostera™ PCV

Two types of circoviruses have been identified in swine, porcine circovirus 1 (PCV1) and porcine circovirus 2 (PCV2), where only the latter is considered pathogenic (124). PCV2 is the causative agent of Porcine Circovirus Associated Disease, which includes multiple clinical syndromes of swine such as Post-weaning Multisystemic Wasting Syndrome, porcine dermatitis and nephropathy syndrome, and PCV2-induced reproductive disorders (125–127).

Ingelvac CircoFLEX® (produced by Boehringer Ingelheim®), Circumvent® PCV-M G2 & and Porcilis® PCV (both produced by Merck), and CircoGard (produced by Pharmgate Biologics) are licensed subunit vaccines that were developed using a BEVS system to express the PCV-2 ORF-2 protein (128). For both Ingelvac CircoFLEX® and Circumvent® PCV-M G2 vaccines, the ORF-2 protein is used as a basis to elicit an immune response in swine against PCV-2 (129). In general, vaccination with these technologies in young piglets resulted in attenuated weight loss, shortened viremia, and lower viral load (130). Fostera™ PCV vaccine produced by Zoetis is single-dose inactivated chimeric PCV1-2 viral-vector vaccine. It utilizes the genome of the non-pathogenic PCV1 as the backbone, cloned with the ORF2 gene of PCV2 which encodes the immunogenic capsid protein of the virus (131). Vaccinated animals demonstrated increased concentration of neutralizing antibodies and anti-PCV2 IgG antibody titers which correlate with the significant reduction of viremia and replication of PCV2 compared to negative control animals (132, 133). Moreover, this chimeric vaccine induced a strong cell mediated immune response (CD3+ and CD4+ cells) that may explain the decrease of PCV2 genomic copies in the blood of immunized pigs (132).

#### Suid Herpesvirus-1 (Pseudorabies/Aujesky's Disease)—Porcilis® Begonia (MSD Animal Health- Intervet), Auskipra® GN (Hipra)

Suid herpesvirus 1 (SuHV-1) is an *Alphaherpesvirus* responsible for Aujesky's disease (also known as Pseudorabies). This highly contagious pathogen infects a wide range of animal species with swine being the principal reservoir and host of the virus. Disease in pigs includes a variety of clinical symptoms, neurological signs and high mortality rate up to 100% in piglets while older pigs mainly showcase respiratory signs. Infected sows demonstrate a variety of reproductive disorders such as abnormal return to estrus, abortions, stillbirth, mummified or week piglets (50). The predominant clinical symptoms in secondary hosts (cattle, dogs, and cats) are severe pruritus and neurological disorders (127). Nonetheless, this pathogen causes significant economic losses in naïve pig farm production sites and still remains a notifiable disease in the USA (134). SuHV-1 is a DNA virus comprised of several genes that contribute to the pathogen virulence but are not essential for viral replication while the tk and gE genes have been the primary target for deletion to achieve inactivation of the virus.

Porcilis® Begonia (MSD Animal Health- Intervet) is a tk and gE deletion mutant live attenuated vaccine. It is being used for the prevention of clinical symptoms and mortality by Aujesky's disease. This vaccine has been developed to protectively immunize the animals for a period of 4 months (135, 136). Auskipra® GN (Hipra) is a live attenuated gE negative Bartha K61 strain vaccine and has shown to prevent clinical symptoms and reduce viral shedding of Chinese SuHV-1 variants (AH02 strain) (137, 138). Both of the vaccines can been used in vaccination programs to control and eradicate pseudorabies (139, 140).

#### Pestivirus—Suvaxyn® CSF Marker, Porcilis® Pesti, and Bayovac CSF E2®

Classical swine fever (CSF), is caused by a *pestivirus* of the family *Flaviviridae* (127). CSF virus (CSFV) is a small, enveloped virus with a single-stranded positive sense RNA genome which encodes a polyprotein, post-translationally cleaved to 12 final products, including the E2 structural glycoprotein that has a critical role in viral replication (141, 142). The eradication of CSF in several countries in Western Europe, North America and Australia is by in large credited to the Chinese lapinized vaccine (C-strain), an attenuated strain of CSF, developed by China Institute of Veterinary Drugs Control and Harbin Veterinary Research Institute in 1956 (143). However, this highly contagious viral disease remains of worldwide significance with a high mortality rate. CSFV is still endemic in many parts of the world, including most of Asia, Central and South America and multiple countries in Eastern Europe, resulting to sporadic outbreaks in highly susceptible naïve swine populations in neighboring CSF free countries (127, 144).

Pigs are typically infected with CSFV by the oronasal route, by contact of susceptible swine with infected feral or domestic pigs, or ingestion of uncooked swill, with tonsil as the initial site of viral replication. Animals in the acute form of disease, are exhibiting high fever, loss of appetite, depression, and conjunctivitis frequently succeeded by diarrhea, vomiting, cutaneous erythema and central nervous system clinical signs, days or weeks before they eventually die. Additionally, CSFV is able to cross the placenta and transmit to the fetuses resulting to mummifications, abortion, stillbirths or fetal deformities (127, 144, 145).

A promising commercially available vaccine is Suvaxyn® CSF Marker, the CP7_E2_alf chimeric vaccine which is licensed by the European Medicines Agency. The vaccine utilizes a live-attenuated bovine viral diarrhea virus (BVDV) backbone a expressing the E2 glycoprotein of CSFV (146). This is an effective strategy as the E2 glycoprotein is the major neutralizing antigen of CSFV (147, 148). In addition, the design of the CP7_E2_alf vaccine enables the serological differentiation between wild-type infected and vaccinated swine in herds (149, 150). Intramuscular (IM) and oral vaccination has been shown to confer full protection against challenge with the highly virulent CSFV strain “Eystrup” 28 days after immunization (146, 149). Challenging vaccinated animals within 2 days after immunization conferred partial protection (151). Additionally, duration of immunity has been shown to last at least 6 months after one vaccination dose (152).

Porcilis Pesti® (Merck) and Bayovac CSF E2® (Beyer AG) are licensed subunit vaccines developed using the BEVS system to express the E2 protein (153). Porcilis Pesti® has shown to be very efficacious against the low virulent strain “Glentorf” in pregnant sows, as no virus was detectable following a vaccination-challenge study and nine out of 10 litters of the vaccinated sows were protected from CSFV infection when challenged 126 days from vaccination and on day 65 of gestation (154). In a large-scale laboratory trail, both Porcilis Pesti® and Bayovac CSF E2®, were evaluated. The data revealed animals vaccinated with Bayovac CSF E2® were better protected against clinical CSF than those that received Porcilis Pesti® as the antibody response was more pronounced and the transmission probability was reduced significantly after the second dose. When sows were challenged with virulent CSF 14 days after vaccination (day 60 of gestation) with Bayovac CSF E2® and Porcilis Pesti®, 75 and 100% of the sows had viremic piglets, respectively (155). This data collectively suggests that these vaccines have reduced efficacy during an emergency field outbreak situation in which animals had not been vaccinated at least 3 weeks prior to exposure.

#### Porcine Endemic Diarrhea Virus –iPED+ Vaccine

Porcine Epidemic Diarrhea virus (PEDv) is a highly contagious swine coronavirus causing enteritis in all age groups with a variable virulence and mortality depending in the strain (156, 157). PEDv is an enteropathogenic coronavirus comprised of a positive sensed RNA genome that encodes a spike (S) glycoprotein located on the outer surface envelope of the virus particle. The spike (S) protein of PEDv is crucial for the virus interaction with host cell receptors and was characterized to contain many epitopes recognized by the host's immune system to incite neutralizing antibodies (158–160).

iPED+ vaccine (updated to iPED RNA) was the first *Alphavirus*-derived replicon RNA particle vaccine licensed to control PEDv. The vaccine employs a replicon vector system which utilizes a defective Venezuelan equine encephalitis virus (VEEV) like particle to deliver and propagate the PEDv S glycoprotein antigen in swine (161, 162). The iPED RNA vaccine was shown to elicit PEDv-neutralizing antibodies in dams and passively acquired PEDv-neutralizing antibodies in suckling piglets, induced clinically protective immunity and reduced viral shedding in challenged pigs, and reduced farrowing mortality in challenged sows (161, 163, 164).

#### Swine Influenza a Virus– SEQUIVITY®

Swine influenza A virus (swIAV) is a major respiratory pathogen in pigs resulting in delayed growth, prolonged finishing time, and consequential economic damage (165–168). Sequivity is a 3rd generation vaccine technology that employs the Sequivity™ RNA Particle Technology, an alphavirus replicon vector system derived from the attenuated TC-83 strain of VEEV (45, 169, 170). This vaccine has not been shown to be efficacious when given in the presence of maternal antibodies but does induce a strong humoral and cell-mediated immune response in animals without maternal antibodies (45, 171–173). Additionally, this vaccine platform allows the option for “Veterinary Prescription” or customized vaccines, similar to autogenous vaccines, in which individualized, single or multivalent formulations can be produced on a case-by-case basis. Accordingly, an immunogenicity and efficacy trial evaluating an H3 RP vaccine showed this vaccine platform elicited protective serologic response within 3 weeks of receiving the boost vaccination, induced a specific IFN-γ response, prevented detectable nasal shedding and live virus within broncho-alveolar lavage fluid, and attenuated clinical disease (173).

### Bovine Vaccines

#### Foot and Mouth Disease—Adt.A24 FMD vaccine

Foot-and-mouth disease (FMD) is caused by a highly contagious *Aphthovirus* that transmits between cloven-hoofed ungulates. The virus is a member of the *Picornaviridae* family and can be transmitted through aerosol droplets, direct contact and/or from ingestion by susceptible animals. On average 11 billion dollars (USD) is lost per annum in countries where FMD is prevalent (174). The devastating global economic impact of FMD has fast-tracked the research into FMD vaccines using novel technologies. Of interest, includes the Adt.A24 FMD vaccine, which was granted conditional licensure by the United States Department of Agriculture (USDA) to protect cattle in 2012 (175). The replication deficient Adt.A24 vaccine utilizes a human *adenovirus* construct as a vector to deliver empty capsids of the A24 FMD strain to elicit an immune response (175). Previous studies in bovine and swine has shown that the Adt.A24 vaccine prevents FMD, along with FMD viremia 7-days after initial vaccination and is most efficacious when combined with the ENABL® adjuvant (176, 177). This vaccine has no reversion to virulence, no shedding from vaccines to naïve animals, no excretion in milk from lactating dairy cattle and conferred 64% efficacy against clinical FMD (178, 179). Lastly, the Adt.A24 vaccine enables the use of a DIVA strategy for evaluating herds during an outbreak.

#### Bovine Herpesvirus Type 1 - Bovilis® IBR Marker Live, Hiprabovis® IBR Marker Live, Bayovac IBR Marker Vivum, Bayovac IBR Marker Inactivatum, Rispoval® IBR-Marker Inactivated, Rispoval® IBR-Marker Live

Cattle infected with Bovine Herpesvirus 1 (BoHV-1) are at risk of developing Infectious Bovine Rhinotracheitis (IBR), an acute and highly contagious disease affecting the upper respiratory tract (180). Additionally, BoHV-1 infection can also impact fertility, reproduction, and productivity. Bovilis® IBR Marker Live, Hiprabovis® IBR Marker Live, Bayovac IBR Marker Vivum, Bayovac IBR Marker Inactivatum, Rispoval® IBR-Marker inactivated, and Rispoval® IBR-Marker live are licensed vaccines for use in cattle against BoHV-1. All of these IBR vaccines have the gE- deletion; the Hipravovis® IBR Marker live also has the tk- deletion. A disadvantage to utilizing some of these modified-live gE-product is the potential for latency in immunized animals and consequent reactivation or shedding following a provoked immunosuppressive state (181, 182). It has been shown that inactivated gE-deleted vaccines reduced viral excretion more efficiently than live gE-deleted vaccines in latently infected animals induced into an immunosuppressive state (183). Nonetheless, these marker vaccines administered either IM or IN induce a robust humoral and cell-mediated immune response making them versatile and valuable (184). Bovilis IBR Marker Live has been shown to prohibit nasal secretion shedding, prevent viremia, to elicit a humoral immune response in pregnant cattle until at least 180 days post calving, and provide passive immunity to calves until at least 180 days post calving (185, 186).

### Poultry Vaccines

#### Avian Influenzas—Trovac®-AIV H5, Vectormune®AI

Avian Influenza Viruses (AIV), are important pathogens for both poultry production and for human health. AIVs are enveloped, negative sense single stranded RNA viruses of the *Orthomyxoviridae* family and are classified as either highly pathogenic or low pathogenicity in avian species. Trovac®-AIV H5 (TROVAC-H5), produced by Boehringer-Ingelheim, contains a live recombinant fowl pox-vectored backbone that expresses an H5 HA subtype isolate synthetically generated based on a highly pathogenic AIV HA protein and altered to mimic a low pathogenicity virus. When a single dose was administered to 1-day old chicks, duration of immunity lasted at least 20 weeks providing significant and rapid protection especially within field conditions (187, 188). Importantly, this vaccine was not efficacious against animals pre-immunized against or infected with fowlpox as protection against AIV levels decreased (189).

Vectormune® AI from CEVA Animal Health, uses a similar synthetic avian IAV HA protein inserted into a turkey herpesvirus (HVT) backbone. Vaccination conferred robust and long-lasting protection in commercial flocks, prevented the development of clinical disease, and suppressed shedding of high-pathogenicity avian influenza (190, 191).

#### Newcastle Disease—Innovax®-ND, Vectormune® FP-ND

Newcastle Disease (ND) is a viral disease of domestic poultry, including chickens, turkeys, pigeons, pheasants, ducks and geese, of a worldwide importance (192). The infectious agent, Newcastle Disease Virus (NDV) or avian paramyxovirus serotype 1, is a highly contagious, negative sense single stranded RNA, virus of the *Paramyxoviridae* family. Transmission of NDV can occur by inhalation or by ingestion of contaminated feed or water, *via* the discharges and droppings of infected birds, and can spread rapidly through the flock. Like AIV, NDV can be further classified on the basis of its virulence, as velogenic (highly pathogenic), mesogenic (moderate pathogenicity), or lentogenic (subclinical or avirulent). Velogenic strains cause acute respiratory disease accompanied by nervous signs and high mortality that in susceptible flocks can approach to 100% (193, 194).

ND has seen advancements in commercial vaccine technologies similar to AIV. Innovax-ND, from Merck, inserts the Fusion (F) protein, a strongly immunogenic antigen, gene from NDV into an HVT vector. As with any HVT vector, vaccinated animals developed strong immunity against MD, but importantly developed protection against lethal challenge with NDV (195). A more recent and novel development in ND vaccines is Vectormune® FP-ND from Ceva which also utilizes a viral vector, however in this case it is Fowl pox.

#### Infectious Bursal Disease, Mareks, Disease and Infectious Laryngotracheitis—Innovax® ND-IBD, Innovax® ND-ILT

Infectious Bursal Disease (IBD) is caused by a double stranded DNA virus (IBDv) from the *Birnaviridae* family. IBD is a highly infectious disease of young domestic chickens and turkeys, characterized by immunosuppression and bursal atrophy due to depletion of B-lymphocytes. While in most cases, IBD-related morbidity is high and mortality is low, certain highly virulent strains can cause up to 60% mortality (196, 197). While IBDv targets B-lymphocytes, Marek's disease (MD) virus (MDV), also called alphaherpesvirus 2 or gallid herpesvirus 2, primarily preys on CD4^+^ T- lymphocytes. MDV is a highly oncogenic lymphotropic virus with worldwide distribution causing lymphoproliferative disease in chickens. Marek's disease is characterized by paralysis due to widespread presence of T-cell lymphomas localized in peripheral nerves, and visceral organs (198). Another important herpesviral disease of poultry is Infectious Laryngotracheitis (ILT), which is caused by the avian Alphaherpesvirus 1 or gallid herpesvirus 1. ILT virus (ILTv) is a double stranded DNA virus transmitted to birds through aerosols and fomites. ILT is an upper respiratory track disease causing significant economic losses due to high mortality rate (up to 70%) (199).

The traditional method for immunization against MD is *via* a turkey herpesvirus-vectored live vaccine, since HVT is subclinical in poultry, and provides strongly cross-reactive antibodies against MD. This style of multi-protective recombinant vaccines has been popularized, as Merck has produced multiple variants based on this technology. Innovax® ND-IBD uses the HVT-vector, modified to include the F gene from NDV and the VP2 surface glycorptein gene from IBDv. When challenged, animals exhibited protection against NDV, IBDV, and of course MDV for up to 60 weeks (200). Another example is Innovax® ND-ILT, which provides protection against NDV and ILTV. This recombinant FPV has been edited to include the F gene and HN gene from NDV, as well as the gB gene from ILTV. The HN construct from NDV encodes the hemagglutinin/neuraminidase proteins, while the gB gene from ILTV encodes the primary surface glycoprotein antigen. Results from vaccine trials showed roughly 70% protection against ILTV, comparable to the traditional inactivated vaccine, in addition to neutralizing immunity against NDV (201).

## Aquaculture

### Salmonid Vaccines

#### Infectious Hematopoietic Necrosis—Apex IHN

In the aquaculture industry, DNA vaccines have seen more success than other fields and continue to be a major field of development (202). As stated previously, DNA vaccines themselves are immunogenic and function as PAMPS and thus eliminate the need for adjuvants (203). One of the major advantages to this technology in fish is the avoidance of adjuvants which have historically been shown to cause severe reactions, such as peritonitis and melanisation of the muscle tissue in fish (204, 205). Apex IHN from Novartis (now Elanco Animal Health) was developed to vaccinate against Infectious Hematopoietic Necrosis Virus (IHNV), a *Rhabdovirus* that causes extensive necrosis of hematopoietic tissue in early life stages and has a high mortality among Salmonids (206). This disease can affect both wild and farmed salmonids resulting in major economic loss. Apex IHN is a DNA vaccine encoding the glycoprotein (G), a major antigen for protective antibodies. Given IM, this vaccine induces both innate and adaptive immune responses in fish and has conferred significant protection in Atlantic salmon, Pacific salmon, and rainbow trout (207–213). Apex IHN vaccination confers a significantly attenuated mortality rate—<3% in vaccinated animals and 99% in control animals–reduces viral spread among cohabitating naïve Atlantic salmon with infected Atlantic salmon, abolishes disease transmission amongst infected Atlantic salmon cohabitating with naïve sockeye salmon, and induces a long-lasting neutralizing antibody titer (214).

#### Pancreas Disease - Clynav

Clynav, produced by Elanco Animal Health, is another recombinant DNA vaccine containing the puK-SPDV-poly2#1 plasmid and codes for several proteins from the salmonid alphavirus subtype 3. This vaccine has been approved in the EU and Norway and is indicated to protect against pancreas disease. This disease has a significant economic burden due to the mortality, reduced growth rates, and reduced meat quality at time of slaughter (215). Fortunately, Clynav protects against weight loss, reduces the prevalence and severity of morphological tissue lesions within the cardia, pancreas and skeletal muscle, and reduces mortality for up to 1 year after vaccination. Additionally, when compared to a traditional monovalent vaccine, Clynav provided significantly higher neutralizing antibody titers, conferred lower viremia, reduced transmission to cohabitating naïve fish, and conferred a significantly higher weight gain post challenge (216). The major criticism of these DNA vaccines is the incorporation rate in the vaccinated subjects. While the incorporation rate is negligible, however it has not been precisely estimated according to manufacturers but modeled on scenarios estimating integration (217).

## Exotic Animals

### Wildlife

#### Rabies—ONRAB®, RaboralV-RG®

During the last 50 years there has been a significant effort to eradicate rabies virus from domesticated companion animals by establishing mandatory vaccination programs. Currently the attention has been focused on wildlife species that are critical for the prevalence of this fatal disease and transmission to humans. According to the annual Center for Disease Control and Prevention (CDC) report about Rabies surveillance in the United States during 2017, 91% of rabid cases involved feral animals mainly bats, raccoons, skunks and foxes (218). This highlights the importance of the development of different vaccine constructs to control and even eliminate the transmission of rabies by immunizing the most susceptible principal reservoir wildlife species. United States, Canada, and Europe have established an Oral Rabies Vaccination (ORV) program, to prevent the spread of rabies to raccoons, foxes, coyotes, wolves and other species that can serve as reservoirs for rabies.

Two types of recombinant vaccines that express the rabies glycoprotein have been used in oral baits to prevent this zoonotic disease. Onrab® by Artemis Technologies Inc. (Guelph, Ontario, Canada) employs a human adenovirus type 5 (Had5) vectored vaccine. Raboral V-RG® utilizes a vaccinia virus as the backbone. ORV baits are produced by Merial Ltd (Athens, GA) and consist of an edible packet that contains the Raboral V-RG®. Administration of this vaccine has led to the eradication of the zoonotic rhabdovirus from 3 European countries (219). This is due to the higher efficacy of the vaccine in red foxes, which are the principal reservoir species in the continent (220, 221). The main objective of this vaccination program is to confer a neutralizing positive titer over 0.05 IU/ml to the targeted animals. All of the vaccinated foxes, 56% of coyotes and 62% of gray foxes have shown protective serum titers after the administration of the ORV baits (220–222). However, other mesocarnivore animals like raccoons and skunks, which are considered the primary carriers of rabies in the USA have demonstrated variable effectiveness on their immunization using ORV baits (223–225). It has been shown that Onrab® vaccine induces better protection on raccoons by inducing humoral response on 74–77% of the animals, instead of the 30% seropositivity achieved after the administration of the ORV baits (226, 227).

### Lagomorphs

#### Rabbit Hemorrhagic Disease - Novibac Myxo-RHD & Novibac Myxo-RHD Plus

The etiological agent of Rabbit hemorrhagic disease (RHD) is a highly virulent *Calicivirus* that is enzootic in rabbit populations worldwide, causing frequent epidemics with significant mortality rate up to 90% in rabbits older than 5 weeks (127, 228). Another important pathogen of this animal species is Myxoma virus which is a member of the *Leporipoxvirus* genus. Myxomatosis is an acute, systemic and often fatal disease of European rabbits characterized by blepharoconjuctivitis, swellings in the eyes, skin and genitals, listlessness and anorexia (229).

Nobivac® Myxo-RHD is a live chimeric bivalent vaccine that uses a Myxoma viral vector expressing the VP60 capsid protein of the classical 009 RHD viral strain. Nobivac® Myxo-RHD Plus contains a second recombinant Myxoma virus with the VP60 protein of the emerged variant MK 1899 (230). Nobivac® Myxo-RHD confers significant protection against both of the pathogens for 12 months after a single dose administration. In an immunization study all of the vaccinated animals seroconverted showing a strong humoral response against RHDV which is essential for the prevention of this viral disease in the challenged animals (231).

## Discussion

Historically, vaccines in human medicine have been in the wake of veterinary medicine as there are very limited licensed approved second and third generation vaccines in human medicine. The hepatitis B vaccine was the first example of a synthetic vaccine developed using recombinant DNA technology and was licensed in 1986; Hemophilus influenza B (HIB), the first conjugate vaccine, was licensed for medical usage in 1987; The Dengue tetravalent vaccine, trade name Dengvaxia, utilizes a live-attenuated tetravalent vaccine consisting of chimeric Dengue proteins combined with the non-structural genes of the Yellow Fever 17D vaccine strain. The rVSV-ZEBOV vaccine against Ebola Zaire, approved in 2019, is a live recombinant viral replication-competent Ebola vaccine consisting of a vesicular stomatitis virus backbone with the envelop glycoprotein of the Zaire ebolavirus in place of the VSV envelop glycoprotein. A heterologous 2-dose vaccination scheme with the Zabdeno (Ad26.ZEBOV) and Mvabea Ebola (MVA-BN-Filo) vaccines are approved for use in the EU. Zabdeno is the prime vaccination and is an adenovirus type 26 vector expressing the Ebola virus Mayinga variant's glycoprotein. MBA-BN-Filo serves as the boost immunization and is a non-replicating, recombinant, modified vaccinia Ankara (MVA) vector-based vaccine encoding glycoproteins from Zaire Ebola virus, Sudan virus, Marburg virus, and the nucleoprotein from the Tai Forest virus respectively (232).

In late 2020, the United Kingdom became the first sovereign country to approve Tozinameran INN, a messenger RNA vaccine (co-produced by Pfizer and BioNTech) indicated for the prevention of SARS-CoV-2 infection, the agent responsible for the COVID-19 pandemic (233). This is the first instance in which a gene-based technology has been licensed and approved for an infectious agent. Since then, and in the midst of the pandemic, other novel and third generation vaccine candidates have been approved for Emergency Use Authorization (EUA) or are undergoing final stages toward EUA application. At the time of writing, these candidates include the Moderna mRNA vaccine, mRNA-1273, and the adenovirus-vectored vaccine AZD1222 by AstraZeneca and Oxford University (234, 235).

Continuing to optimize delivery systems, and to enhance mucosal immunity, molecular adjuvants are crucial for the synergism of vaccine development. However, to remain within the scope of licensed novel technologies in veterinary medicine, the aforementioned components will only be briefly discussed as many are still in experimental stages.

One technology in which human medicine has arguably preceded veterinary medicine is the employment of viral-like particles (VLPs). VLPs are non-infectious/void of genetic material, self-assembling complexes that bear antigens of interest and mimic the overall structure of a virus (236). The VLP technology has seen success as it activates the adaptive immune response *via* both MHC-I and MHC-II complexes and are consequently capable of stimulating robust CTLs and CD4+ T helper cells (237). Vaccines against human papillomavirus (Cervarix®, Gardasil®, and Gardasil9®) and Hepatitis B virus (Sci-B-Vac™) also utilized the recombinant technology assembled onto a virus-like particle (VLPs) (238).

Nanoparticles (NPs), similar to VLPs, are a revolutionary delivery technology widely investigated for therapeutic drugs and vaccines. Characterized for their size (<100 nm), several types of NPs composed of gold, dendrimers, carbon polymers, an liposomes have been shown to improve vaccine efficacy, facilitate antigen uptake, and induce desired immunological responses (239). NPs offer several advantages: they can directly access lymphatic drainage systems for immune processing, can be modified to target specific subsets of immune cells, and can be delivered to specific intracellular compartments to hone in on specific immune pathways (240). As such, much of the success of the mRNA SARS-CoV-2 vaccine platform was the use of lipid NPs (241). Nonetheless, a comprehensive understanding of how NPs can be utilized to optimize vaccine delivery remains and many experimental NP candidates are currently being explored in clinical trials for influenza (NTC032293498, NCT3658629), and respiratory syncytial virus (NCT01960686, NCT02247726, NCT02624947) vaccines (240).

Some of the major gaps in vaccine development are the elicitation of mucosal immunity *via* induction of secretory IgA and the appropriate immune stimulation to the antigen *via* adjuvants. The vast majority of pathogens gain entry into hosts *via* mucosal sites, yet the majority of current vaccines provide partial or no protection at mucosal sites. In veterinary medicine, mucosal vaccines have been more successful as sprays and drinking water vaccines are routinely utilized, however, there are no licensed human vaccines for mucosal-transmitted pathogens (242). Vaccine-induced mucosal immunity is particularly challenging due to the difficulty in protecting and preserving antigen structural integrity and increasing the bioavailability of mucosal vaccines. Some experiments have seen success with the use of nanoparticle formulations by incorporating polyethylene glycol (PEG) (243). Chitosan, a non-toxic polymer has also been utilized in intranasally delivered Escherichia coli O157:H7 vaccine formations with similar success (244). Immunostimulating complexes (ISCOMs) are spherical cage-like experimental adjuvants composed of phospholipids, cholesterol, saponin, and protein antigens and have been particularly successful in mucosal immunizations resulting in secretory IgA and systemic immune responses (245, 246). This technology has been utilized in the equine influenza vaccine Equip ™ F (produced by Zoetis/Pfizer Animal Health), a subunit vaccine shown to stimulate both humoral and cell-mediated immunity (247, 248).

A promising solution to combat poor immunogenicity, for DNA vaccines specifically, are molecular adjuvants. These generally comprise plasmid-encoded signaling molecules such as cytokines, chemokines, and immune costimulatory molecules, but newer approaches include gene knockdown and systems biology (249–251). For example, Interleukin-2 (IL-2) promote differentiation of naïve T cells into effector cells and facilitates the generation of memory T cells (20). Thus, IL-2 has been one of the most extensively studied molecular adjuvants and has shown increased immunogenicity for previously low-immunogenic vaccines such as HIV, influenza, and SARS-CoV (252–255). Other immunomodulatory cytokines being evaluated as molecu lar adjuvants are IL-15, IL-12, and MG-CSF (250, 252).

The evolution of vaccine technologies mirrors the continued and rigorous advancement toward safe, efficacious, stable, and cost-effective vaccines for existing and emerging infectious pathogens. Veterinary medicine continues to trail blaze the path as evident by the numerous novel technologies already employed.

## Author Contributions

VA, VP, PN, JN, and KM researched data for the article and substantially contributed to the discussion of content. VA, VP, and SG drafted and generated figures for the article. VA and CK wrote, reviewed, and edited the manuscript before submission. All authors contributed to the article and approved the submitted version.

## Conflict of Interest

The authors declare that the research was conducted in the absence of any commercial or financial relationships that could be construed as a potential conflict of interest.
